# Quality of medical training and emigration of physicians from India

**DOI:** 10.1186/1472-6963-8-279

**Published:** 2008-12-30

**Authors:** Manas Kaushik, Ananya Roy, Anand A Bang, Ajay Mahal

**Affiliations:** 1 Departments of Nutrition and Epidemiology, Harvard School of Public Health, 677 Huntington Ave, Boston, MA 02115, USA; 2 Department of Environmental Health Sciences, University of Michigan School of Public Health, 6655 SPH I, Ann Arbor, MI 48109, USA; 3 SEARCH, Shodhgram P.O. District Gadchiroli, Maharashtra 442606, India; 4 Department of Global Health and Population, Harvard School of Public Health, 677 Huntington Ave, Boston, MA 02115, USA

## Abstract

**Background:**

Physician 'brain drain' negatively impacts health care delivery. Interventions to address physician emigration have been constrained by lack of research on systematic factors that influence physician migration. We examined the relationship between the quality of medical training and rate of migration to the United States and the United Kingdom among Indian medical graduates (1955–2002).

**Methods:**

We calculated the fraction of medical graduates who emigrated to the United States and the United Kingdom, based on rankings of medical colleges and universities according to three indicators of the quality of medical education (a) student choice, (b) academic publications, and (c) the availability of specialty medical training.

**Results:**

Physicians from the top quintile medical colleges and of universities were 2 to 4 times more likely to emigrate to the United States and the United Kingdom than graduates from the bottom quintile colleges and universities.

**Conclusion:**

Graduates of institutions with better quality medical training have a greater likelihood of emigrating. Interventions designed to counter loss of physicians should focus on graduates from top quality institutions.

## Background

Developing countries continue to lose substantial numbers of trained physicians to developed countries[1]. In light of growing awareness of the adverse impact of physician movement, a few developing countries have asked destination countries to financially compensate the countries of origin for losses [2] and a few developed countries have proposed ethically appropriate methods of recruiting physicians[3]. These efforts notwithstanding, interventions by developed countries to prevent this 'brain drain' are likely to be limited by their need for physicians, along with concerns about protecting physicians' right to mobility. Given the likely small potential impact of existing measures to address the potential impacts of emigration on health service delivery, developing countries need to understand and identify characteristics of migrating physicians that can help better target strategies to prevent loss of physicians due to emigration[1].

Though physician 'brain drain' has been long recognized, designing interventions to address it, such as mechanisms of funding of medical education, and establishment of new medical schools, has been constrained by a limited understanding of this phenomenon, usually stemming from the unavailability of detailed data. Some studies have focused on qualitatively assessing the reasons for physician emigration[4,5] while others studies have documented the extent of migration. Recently, Mullan combined information from medical registers in individual countries to quantitatively assess the magnitude of migration using "emigration factors" – the ratio of the absolute numbers of migrating physicians to the total number of registered physicians in the home country[6]. However, the usage of aggregate emigration factors can obscure the true impact of physician migration on sending countries, if source countries are relatively better off in terms of physician supply, or human development performance[7]. The use of aggregate emigration factors can also be misleading if the individuals who emigrate are better trained and thus, embody higher levels of human capital, than those who remain[8]. Understanding the quality of physicians who join the work-force is also important for developed countries, given patients' and policymakers' concerns about the quality of physicians trained in developing countries[9,10].

We assess whether the quality of medical training received in India is related to physician emigration to the United States and the United Kingdom for individuals graduating between 1955 and 2002 from Indian medical colleges. India offers a particularly interesting case for assessing the linkage between the quality of medical training and the likelihood of emigration. It has a large number of medical colleges managed by a diverse set of authorities, ranging from entities overseen by the central government, various state governments and municipal corporations to private trusts, with considerable variation in sources of funding and budgets. Institutions of "national importance" such as the All India Institute of Medical Sciences (AIIMS) are funded primarily from central government budgets and there are medical colleges heavily subsidized by state and municipal governments. Medical colleges operate by private trusts, which have seen a rapid expansion in the last three decades, are funded mainly by student contributions. The generally lax regulatory environment within which this recent rapid expansion has occurred, has also allowed for considerable inter-college heterogeneity in terms of teaching faculty, access to hospitals, and academic standards for incoming and graduating students[11,12]. To our knowledge, this is the first study linking emigration of physicians with the quality of medical training received by them.

## Methods

### Study setting and data

We compiled data on medical colleges in India and the number of physician emigrants from India from various sources. Information about Indian medical colleges was collected from the official website of the Indian Ministry of Health, websites of medical teaching institutions, state health universities and published sources. Data on the name, location, year of establishment, enrollment capacity for MBBS and various specialty training courses offered was collected[13,14]. Using this information, together with the year a college was founded, and assuming that the number of seats was equal to the number of graduating students, we estimated the total cumulative number of medical graduates produced by each medical college, from 1955, until the end of 2002. This assumption is reasonable, given that the heavy demand for medical seats in India is unlikely to leave any medical college training slots unfilled. The numbers we estimated is only about 2.9 percent lower than the total number of physicians ever registered with MCI[15].

We used the American Medical Association (AMA) master-file of physicians and General Medical Council's physician register to calculate the cumulative number of Indian physicians.

### Measures of quality

Though various periodicals provide ranking of medical schools, there is no agreement about transparent, objective and quantifiable methods of ranking institutions [16]. We used three different indicators of the quality of training received at a medical college (see additional file 1). First, we used a bibliometric index to assess the quality of medical colleges that has been previously used to rank individuals, faculties, universities and countries [17-19]. The ranking of 107 medical colleges, established prior to 1980 in India, by this method has been published previously[20]. Briefly, the number of publications was abstracted from Science Citation Index (SCI) produced by medical colleges over a 9-year period from 1980–88, with a greater number of publications (per undergraduate admission seat) in peer-reviewed journals as an indicator of better faculty and medical training. This ranking was applicable to both public and privately-operated medical colleges, as well for certain public sector institutions that were omitted in the student preference ranking. Because more updated information on publications was unavailable, the ranking excluded many private medical colleges established after 1980, some of which been the subject of quality concerns[12].

We ranked medical colleges by the size of their specialty (M.D/M.S equivalent of residency) and sub-specialty training programs (M.Ch/D.M, equivalent to fellowships in USA). This ranking captures the assessment of infrastructure and the faculty by Medical Council of India (MCI) – the accreditation and regulatory authority of medical education in India[12]. This information was available for all 163 Indian medical colleges that existed in 1997 (the year when the last cohort of the graduates included in our study was admitted to medical college), and is the most comprehensive of all the indicators used here. All three of the ranking procedures were strongly positively correlated. For instance, the correlation coefficient of the student preference-based ranking with the publications based ranking was 0.70.

Finally, we relied on students' own assessment of quality of medical colleges as expressed when they choose medical colleges to pursue education. The Central Board of Secondary Education (CBSE) in New Delhi conducts an India-wide pre-medical test (AIPMT) each year for admission to about 15 percent of all available seats in MBBS (Bachelor of Medicine and Bachelor of Surgery) courses offered in public sector-operated medical colleges. Students indicate their college preferences, and depending on their exam-ranking and seat availability, are given their first choice of college; otherwise they get a college for which they indicated a lower preference. Because higher-ranked students are allowed the first pick, colleges to which students ranked lower in the exam are admitted likely indicate lower perceived quality compared to those admitting higher ranked students.

We believe that the student-preference based ranking is a unique, transparent and composite indicator of student perceptions of quality of medical training and access to facilities. Colleges not participating in AIPMT account for only about 2.5 percent of all public-sector medical college seats in India (authors' calculations). Using the student preferences in the 2007 AIPMT exam, we ranked 92 medical colleges in the public sector that graduated students between 1955 and 2002.

### Analytical strategies

We calculated emigration fractions (ratio of cumulative numbers of emigrant physicians to the cumulative numbers of medical graduates) for the period 1955 to 2002 for Indian physicians emigrating to the United States and the United Kingdom separately, as well as for both countries combined.

We calculate the emigration fraction to the United States, by colleges, ranked in quintiles of three indicators of the quality of medical training. To calculate the emigration fraction to the United Kingdom and combined UK and USA, information for individual medical colleges was consolidated at the level of universities, with which the colleges were affiliated. This was done because the medical register maintained by the General Medical Council of the United Kingdom provided information only on the university from which the physicians graduated, not the individual medical college. The process of consolidating information on emigration and seats from individual colleges to universities to construct university-level emigration fractions was complicated by the fact that during 1955 and 2002, some universities were renamed and others reorganized in India. This process occurred only slowly prior to the mid-1980s, with a few large universities being either renamed or broken up into smaller universities. Subsequently, after a national debate in 1983 on the standards of health sciences education, new universities were established in some states[21]. Thus, some medical colleges' affiliations to universities changed over time. New private medical colleges, whose admissions are not linked to the national AIPMT exam, were also affiliated to these universities.

To construct university-level quintile rankings of the quality of training, universities were scored on the basis of the average (weighted) scores of their constituent colleges, for each of the indicators of the quality of training. As a first step, we limited ourselves to graduates and emigrants for the period before 1985, thereby mitigating the complications introduced by the post-1985 reorganization of universities in southern and western Indian states. We extended our analysis of emigration fractions to 2002 by ranking universities in the post-1985 period on the basis of the same quintile classification range as for the pre-1985 period. The corresponding numbers of medical graduate and emigrants in the post-1985 period was estimated, and the two groups (pre-1985 and post-1985) of graduates and emigrants were then combined to calculate the overall emigration fractions for the period 1955–2002.

Given that information on student preferences and publications was unavailable for many private colleges and some public medical colleges, we assumed that colleges with missing information had the same weighted mean student preference score as the ones for which information was available, provided the university affiliation was the same. With the majority of colleges with missing information being privately run, many being of lower quality than public facilities, this assumption likely biases our results towards the null. Moreover, the consolidation of colleges into universities mixes up both high- and low-ranked medical colleges in the same pool, which would also tend to bias our results towards the null. On the other hand, the fact that each university has common written and practical exams for students in affiliated colleges, suggests that standards for trained graduates are still likely to differ across universities. We found that no college belonging to the top-quintile of colleges mapped onto a bottom-quintile university and no bottom-quintile college mapped to a top-quintile university, suggesting that college rankings translate reasonably well into ranking universities.

We used frequency tables and chi-square tests to statistically assess the association between emigration fractions, and indicators of the quality of training.

## Results

An estimated 575,000 students graduated from Indian medical schools between 1955 and 2002, about 80% of who were graduates of publicly funded medical colleges. Over the same period, 47,527 physicians, trained in India, were registered in the AMA master-file. Of these 46,083 were born outside of the United States and the United Kingdom, and taken as the "emigrants." In the GMC database, 26,655 physicians graduated from Indian medical schools during this period. This yields an overall physician emigration fraction for migration to the United States and the United Kingdom of 12.6 percent between 1955 and 2002.

In table 1, we report our estimates of emigration fractions for Indian physicians to the United States between 1955 and 2002, broken down by quintile ranking of the college of graduation. Emigration fractions of medical graduates in the top quintile of college ranks were two to four times as high as emigration fractions for graduates from the bottom quintile ranks of colleges. Individuals graduating from colleges with relatively larger post-graduate programs had higher emigration fractions for the United States than colleges with smaller post-graduate programs.

**Table 1:** Physician emigration fraction to the United States, by college (quintile) rank

**Ranking/Quintiles weighted by Seats**	**Emigration Fraction (As % of Total Graduates)**		
	
	**Specialty and Sub-Specialty Seats per Undergraduate**	**Student Choice-Based Ranking**	**Publications per Undergraduate**
**I(lowest)**	2.93 (2.80, 3.06)	2.45 (2.32, 2.58)	5.94 (5.78, 6.11)

**II**	4.93 (4.78, 5.07)	5.11 (4.94, 5.28)	6.70 (6.55, 6.84)

**III**	7.42 (7.29, 7.55)	5.47 (5.32, 5.62)	6.58 (6.44, 6.72)

**IV**	8.72 (8.59, 8.85)	7.72 (7.56, 7.88)	8.56 (8.40, 8.71)

**V(highest)**	9.39 (9.24, 9.54)	9.60 (9.43, 9.77)	10.22 (10.04,10.39)

**Total**	7.46 (7.39, 7.52)	6.74 (6.66, 6.81)	7.70 (7.63, 7.77)

**Number of Colleges**	**163**	**92**	**107**

*Note*: College quintile ranks range from I (the lowest) to V (the highest). Quintile rankings of specific colleges are dependent on the method of ranking used; Mantel-Haenszel Chi-Square test for trend p value < 0.0001; 95% confidence intervals are indicated in parentheses. Student choices were available for 93 public sector medical colleges, of which 92 were founded before 1998; Peer-reviewed publications data were available for 107 medical colleges (public and private); and specialty and sub-specialty seat data for all medical colleges, public or private that existed in 1997, the year of admission for last cohort of graduates included in our study.

**Figure 1:**
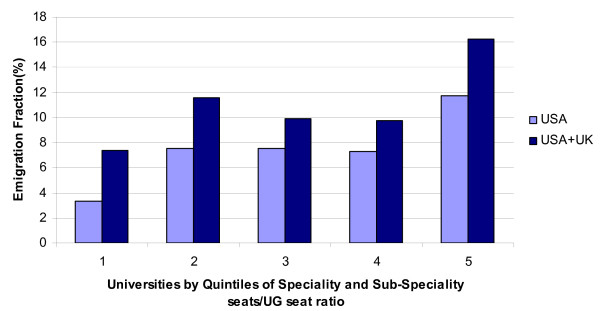
Quality of Medical Education and Migration of Physicians from India

Table 2 presents the results of our analysis of Indian physician migration to the United States and United Kingdom, using university rankings based on publications and student preferences. These results are reported separately for the period before 1985, to take account of the reorganization of universities in India, as well as for the entire study period, from 1955 to 2002. 2.16% of graduates of lowest ranked university on student choice immigrated to USA compared to 8.67% graduates in the highest ranked university. Similarly, 1.89% of graduates of lowest ranked university on student choice emigrated to UK compared to 5.54% graduates in the highest ranked university. The number of medical graduates emigrating to USA, and UK from the lowest ranked university on number of publications per undergraduate seat was roughly half the number of medical graduates emigrating from the highest ranked universities (table 3). Figure 1 depicts the combined emigration fractions to the United States and the United Kingdom by university rankings based on specialty/sub-specialty seats per undergraduate is consistent with these findings.

**Table 2:** Physician emigration fraction, by university (quintile) rank

	**Emigration Fraction (Based on Student Choice-Based Ranking) USA**				**Emigration Fraction (Publications per Undergraduate)**			
	**USA**		**UK**		**USA**		**UK**	
**Ranking/Quintiles weighted by Seats**	**Overall**	**Graduates Before 1985**	**Overall**	**Graduates Before 1985**	**Overall**	**Graduates Before 1985**	**Overall**	**Graduates Before 1985**
**I(lowest)**	2.16 (2.04, 2.29)	2.50 (2.35, 2.66)	1.89 (1.77, 2.00)	1.45 (1.32, 1.57)	5.05 (4.87, 5.22)	5.72 (5.48, 5.96)	2.66 (2.53, 2.79)	1.34 (1.22, 1.47)
**II**	4.02 (3.87, 4.18)	3.62 (3.42, 3.82)	5.85 (5.66, 6.03)	2.14 (1.99, 2.30)	5.02 (4.87, 5.17)	5.95 (5.76, 6.14)	2.17 (2.06, 2.27)	2.00 (1.88, 2.12)
**III**	8.18 (8.02, 8.35)	8.63 (8.44, 8.82)	7.35 (7.20, 7.51)	5.16 (5.00, 5.32)	7.78 (7.66, 7.90)	6.89 (6.73, 7.05)	6.37 (6.26, 6.48)	4.88 (4.74, 5.02)
**IV**	8.85 (8.68, 9.01)	9.79 (9.57,10.00)	6.64 (6.49, 6.78)	6.29 (6.11, 6.47)	7.90 (7.75, 8.04)	8.60 (8.42, 8.77)	4.39 (4.28, 4.51)	3.06 (2.95, 3.17)
**V(highest)**	8.67 (8.47, 8.86)	7.99 (7.77, 8.22)	5.54 (5.37, 5.71)	3.02 (2.87, 3.17)	11.23 (11.05,11.41)	10.97 (10.77,11.18)	5.69 (5.55, 5.83)	4.59 (4.44, 4.73)
**Total**	7.08 (7.01, 7.16)	7.41 (7.32, 7.51)	5.89 (5.82, 5.97)	4.16 (4.09, 4.24)	7.85 (7.78, 7.92)	8.04 (7.95, 8.12)	4.81 (4.75, 4.86)	3.55 (3.49, 3.61)
**Number of Universities**	**56**	**52**	**56**	**52**	**64**	**63**	**64**	**63**

*Note*: University quintile ranks range from I (the lowest) to V (the highest). Quintile rankings of specific Universities are dependent on the method of ranking used; the rank of a university is mapped from information for its constituent colleges on the relevant criterion (publications, student choices, or specialty and sub-specialty seats per undergraduate student). Because of the re-organization of universities after 1985, and some break-up of universities into smaller ones prior to 1985, university-wise quintile rankings were constructed as follows. First, the "original" undivided universities prior to 1985 were ranked in quintiles, separately by each of the two criteria (publications and student preference-based choices). The number of medical college graduates and emigrants up to 1985 were assigned to these quintiles, and the appropriate emigration fractions calculated. This yielded the emigration factors in columns 3 and 5. For calculating "overall" emigration fractions for universities over the entire 1955–2002 period (reported in columns 2 and 4), we ranked universities in the post-1985 period using the same classification range as for the pre-1985 period, and calculated the corresponding numbers of medical graduate and emigrants in the post-1985 period. The two groups (pre-1985 and post-1985) of graduates and emigrants were then combined to calculate the overall emigration fractions for the period 1955–2002. Mantel-Haenszel Chi-Square test for trend p value < 0.0001; 95% confidence intervals are indicated in parentheses. Student preference-based choices were available for 93 public sector medical colleges, of which 92 were founded before 1998; Peer-reviewed publications data were available for 107 medical colleges (public and private); and specialty and sub-specialty seat data for all medical colleges, public or private.

The emigration fraction (F) was calculated as $F=\frac{E_{US}}{G-E_{UK}}$, where *E*_*US *_is the cumulative number of graduates who migrated to the United States, G is the cumulative number of medical graduates and *E*_*UK *_is the cumulative number of medical graduates who migrated to the United Kingdom.

**Table 3:** Total physician emigration fraction for USA and UK for 1955–2002, by university ranking

**Ranking/Quintiles**	**Emigration Fraction (%)**	
	
	**Based on Student Choice-Based Ranking**	**Publications per Undergraduate**
**I(lowest)**	3.89 (3.73, 4.05)	7.21 (7.01, 7.41)

**II**	9.02 (8.80, 9.23)	6.78 (6.61, 6.95)

**III**	13.45 (13.26,13.64)	11.06 (10.90,11.22)

**IV**	13.45 (13.26,13.64)	11.06 (10.90,11.22)

**V(highest)**	12.53 (12.30,12.75)	14.74 (14.55,14.93)

**Total**	11.50 (11.41,11.59)	11.32 (11.24,11.40)

**Number of Universities**	**56**	**64**

*Note*: University ranking method same as Table 2. The emigration fraction (F) was calculated as $F=\frac{E_{UK}+E_{US}}{G}$, where *E*_*UK *_is the cumulative number of graduates who migrated to the United Kingdom, *E*_*US *_is the cumulative number of medical graduates who migrated to the United States, and G is the cumulative number of medical graduates.

## Discussion

Our study posits that the medical education system plays a key, quantifiable role in migration of physicians[22]. In this study, we found that the quality of medical education, assessed using three criterions, was associated with migration of physicians from India. Based on student preferences and publications, individuals graduating from higher-ranked medical colleges in India had higher emigration fractions vis-à-vis migration to the United States. Individuals graduating from colleges with relatively larger post-graduate programs had higher emigration fractions for the United States than colleges with smaller post-graduate programs. The results were similar when we assessed emigration by [23]ranking of universities. These results did not change substantially when the colleges were aggregated into universities suggesting the robustness of our results.

Descriptive studies from Africa suggest that a small number of institutions contribute disproportionately to the ranks of emigrant physicians in developed countries [23-25]. Using cross-country ecological comparisons within Africa, Arah et. al found that emigration of physicians from Africa is higher from more developed countries and countries with better training infrastructure compared to less developed countries[7]. Higher quality of medical education in countries with better training infrastructure is one possible and unexplored explanation of these results. Our findings indicate a challenge faced by developing countries in retaining high quality human resources for health as they try to improve the quality of health care through better medical education[23].

Better quality education, particularly interaction with specialists and sub-specialists, could promote an interest in advanced training and, thus driving physician movement abroad[4]. It is also possible that better quality institutions have a teaching and training curriculum more aligned with more developed medical facilities, thus preparing them better for working in developed countries than in their home countries[26]. There are other potential explanations of our findings. They could result from the association between the quality of medical education and the resulting ability of students to pass professional exams required for practicing medicine in developed countries. Prior information, informally obtained by recipient country institutions by way of interactions with students and faculty from source countries, about the quality of medical training received in different medical colleges may also influence the acceptance rate of physicians in training programs. The quality of medical training received may also underpin a desire for acquiring more advanced training abroad, an important driver of physician migration[4]. In principle, alumni network connections could confound our results[22], although the difference in emigration fractions of graduates in different colleges is evident even in the first 10 years of their date of establishment. Higher emigration fractions among graduates of higher ranked colleges could also result from the higher economic status of incoming students, with the financial capacity to study abroad. It is also possible that objectively better quality students from amongst the pool of medical students are more likely to migrate without any impact of medical colleges [27]. Nonetheless, these factors are unlikely to constitute a full explanation of our findings. In particular, students who qualify to study at government-run medical colleges in India are chosen from amongst thousands of potential candidates who appear in a pre-medical examination, and thus likely to be of high quality. More than 200,000 candidates took the AIPMT examination in 2007, for roughly 1,640 seats[28]. Because expenses in government medical colleges are highly subsidized, economic considerations may be less relevant for admissions at least at government medical colleges, which accounted for 80 percent of all emigrating physicians.

Since we lack detailed longitudinal information on individuals, we cannot disentangle individual-specific factors from institutional factors that drive international migration among Indian physicians. Each medical school fulfills various missions including teaching, research, and patient care and because institutional priorities, capacity and approaches vary, ranking all schools based on a single criterion is of limited utility[29]. We avoided this pitfall by considering three different indicators covering accreditation, research activity and a proxy of faculty input. Nonetheless, we could not consider other methods in evaluation medical schools such as impact on students, alumni giving and public service because of lack of data. Another limitation, stemming from the indicators of quality of medical training we used, is the appropriateness of recent rankings to adequately capture past quality of education provided at these medical colleges. In practice, however, the ranking of colleges and universities on the basis of the number of specialty and sub-specialty seats as well as publications are likely a "cumulative" indicator of past quality, since building infrastructure, both faculty and facilities, take time. Additionally, our method of ranking colleges and universities by quintiles is unlikely to be very sensitive to small changes in individual college, or university, scores.

If the likelihood of physician migration is the same across training institutions, losses in human capital due to emigrating physicians can be addressed by adding to training capacity and additional private sector training institutions might emerge to meet 'physician export' needs[30]. However, as our findings indicate, because the physicians who emigrate are concentrated at higher quality institutions, losses in human capital resulting from emigration cannot be readily overcome by simply expanding private medical colleges. Even though India has expanded medical education rapidly through 'privatization' and trains large number of physicians in recent decades, only a few medical schools have the necessary resources to adequately train future academic and physician leaders. If the well trained group, developed at enormous public expense, is lost permanently, the resulting loss of future leadership in the health sector can adversely affect teaching, and possibly access to good quality health care both in the public and the private sector[31].

## Conclusion

To the extent that higher quality training is supported by greater public subsidies, as is the case with higher education in many developing countries, the disproportionate emigration of well-trained human capital can impose a significant financial burden on those remaining in the home country. These subsidies in higher education may have allowed India to capitalize on greater global inter-linkages, and benefited migrating physicians, but in a resource-constrained environment, this migration likely implies a significant adverse impact on the quality of health service delivery. In these circumstances, a strategy of linking the provision of the public subsidy to the individual medical graduate's decision to migrate is an obvious policy option worth pursuing. However, financial (dis)incentives can only be one element in a mix of methods to keep highly trained individuals. It may require developing country governments, at least the ones that can afford to do so (such as China and India), to set up and expand high-quality research and policy institutions to engage talented individuals. And, it may also necessitate admissions strategies to public medical colleges that emphasize specific lengths of service in the home country.

## Competing interests

MK, AAB, AR are alumni of medical schools in India.

## Authors' contributions

MK and AM collected data, designed study and wrote manuscript. MK and AR analyzed the data. AAB contributed intellectual content to the manuscript. All authors read and approved the final manuscript.

## Pre-publication history

The pre-publication history for this paper can be accessed here:



## Supplementary Material

Additional file 1 **Ranking the quality of undergraduate medical education in India: an overview.** Click here for file
