# Supplementary material for: Quality of medical training and emigration of physicians from India
Source: BMC Health Serv Res. 2008 Dec 30;8:279. doi: 10.1186/1472-6963-8-279 (PMC2637864; doi:10.1186/1472-6963-8-279)
Supplement: Additional file 1 — Ranking the quality of undergraduate medical education in India: an overview. [file 1472-6963-8-279-S1.doc]

**Additional File 1**

**File format: DOC**

**Title: Ranking the quality of undergraduate medical education in India: an overview**

**Description: Brief overview of the methodology used to rank medical colleges in India.**

| **Institution** | **Method of Ranking** | **Formula Used to Construct Ranking** | **Observations** |
| --- | --- | --- | --- |
| **Colleges** | Student Preferences | Mean Examination Ranking of students choosing college in 2006. | Limited to 92 out of 121 public medical colleges.  Private medical colleges; autonomous medical colleges; medical colleges from Andhra Pradesh and Jammu and Kashmir are not included. |
|  | Publications | Faculty Publications in Peer-Reviewed Journals during 1980-88 divided by Number of Undergraduate Seats in the College | Includes both public and private medical colleges; Limited to peer-reviewed publications in 1980-88; Ranking excludes medical colleges founded after 1988. |
|  | Graduate-level Specialty/Sub-Specialty Programs | Total number of graduate-level specialty and sub-specialty seats divided by the number of undergraduate seats in each college | Ranking covers all medical colleges, public and private |
| **Universities** | Student Preferences | Mean Examination Ranking of students choosing college affiliated to the University in 2006 | Ranking covers 56 universities. Ranking based on information on 92 affiliated public colleges for which student preference information was available. |
|  | Publications | Faculty Publications in Peer-Reviewed Journals during 1980-88 divided by Number of Undergraduate Seats in the University | Ranking covers 64 universities. Ranking based on information on 130 affiliated colleges for which faculty publication information was available for 1980-88 |
|  | Graduate-level Specialty/Sub-Specialty Program | Total number of graduate-level specialty and sub-specialty seats divided by the number of undergraduate seats in the University | Ranking covers 79 Indian universities |
